# Transmission dynamics of a novel HIV/AIDS model through a higher-order Galerkin time discretization scheme

**DOI:** 10.1038/s41598-023-34696-6

**Published:** 2023-05-08

**Authors:** Attaullah, Kamil Zeb, Ilyas Khan, Riaz Ahmad, Sayed M. Eldin

**Affiliations:** 1 Department of Mathematics and Statistics, Bacha Khan University, Charsadda, 24461 Pakistan; 2 Department of Mathematics, College of Science Al-Zulfi, Majmaah University, Al-Majmaah, 11952 Saudi Arabia; 3 Department of Mathematics and Statistics, Nanjing University of Information Science and Technology, Nanjing, People’s Republic of China; 4 Center of Research, Faculty of Engineering, Future University in Egypt, New Cairo, 11835 Egypt

**Keywords:** Diseases, Medical research

## Abstract

There are numerous contagious diseases caused by pathogenic microorganisms, including bacteria, viruses, fungi, and parasites, that have the propensity to culminate in fatal consequences. A communicable disease is an illness caused by a contagion agent or its toxins and spread directly or indirectly to a susceptible animal or human host by an infected person, animal, vector, or immaterial environment. Human immunodeficiency virus (HIV) infection, hepatitis A, B, and C, and measles are all examples of communicable diseases. Acquired immunodeficiency syndrome (AIDS) is a communicable disease caused by HIV infection that has become the most severe issue facing humanity. The research work in this paper is to numerically explore a mathematical model and demonstrate the dynamics of HIV/AIDS disease transmission using a continuous Galerkin–Petrov time discretization of a higher-order scheme, specifically the cGP(2)-scheme. Depict a graphical and tabular comparison between the outcomes of the mentioned scheme and those obtained through other classical schemes that exist in the literature. Further, a comparison is performed relative to the well-known fourth-order Ruge–Kutta (RK4) method with different step sizes. By contrast, the suggested approach provided more accurate results with a larger step size than RK4 with a smaller step size. After validation and confirmation of the suggested scheme and code, we implement the method to the extended model by introducing a treatment rate and show the impact of various non-linear source terms for the generation of new cells. We also determined the basic reproduction number and use the Routh-Hurwitz criterion to assess the stability of disease-free and unique endemic equilibrium states of the HIV model.

## Introduction

History shows that infectious diseases can cause havoc in the human population. Although epidemic control has made great strides, infections were thought to be eradicated soon, but they were not. The effect of contagious diseases on society is predicted to be one-fourth of all fatalities worldwide^1^. Some infectious diseases, known as communicable diseases, can be spread from human to human, from human to animal, or from animal to human. The HIV infection is a communicable disease that is among the most devastating and a serious public health issue, and more than 37.9 million individuals are infected worldwide^1^. Infection with HIV damages CD4 + T cells, which are the most essential components of the immune system. The virus progressively weakens the human immune response, making the infected person susceptible to diseases. HIV can be transmitted from HIV-infected people through bodily fluids like blood, vaginal fluids, pre-seminal fluids, sperm, breastfeeding (which can pass HIV from the mother to her infant), sexual activity, and sharing injectable medication equipment like injectors with HIV positive people. HIV infection progresses to AIDS, which is the most severe and chronic phase of the infection^2^. Currently, no effective medication or vaccine exists to cure AIDS, but it can be managed with adequate medical care, such as antiretroviral therapy (ART), which improves health and life while lowering the chance of recurrence. In 2018, the number of individuals living with HIV/AIDS and deaths worldwide is expected to hit 37.9 million and 1.2 million, respectively. Approximately 62% of those infected were confirmed and started on ART^3^. Many therapies have been proposed to improve the health and quality of life of patients infected with HV, including ART^4^, chemotherapy, and stem cell therapy. The combination of drugs in an ART is mostly used to treat HIV infection, which has numerous side effects^5^. Stem cell therapy is very limited due to the high cost of the procedure as well as the difficulty of obtaining healthy and consistent donors. The media may play a critical role in enhancing public knowledge about AIDS infection by persuading individuals to take preventive precautions. In this technological environment, social media platforms are effective tools for spreading awareness about infectious diseases and preventative care.

Mathematical modelling of biological systems is an intriguing area of study that has piqued the interest of a significant number of researchers. A mathematical model is a representation of a dynamical system based on mathematical principles. It is significant in forecasting, evaluating, and regulating HIV infections and several other disease dynamic systems. Several assumptions and parameters have significant consequences for constructing a model utilising controlling functions. Thus, using the idea of optimal control theory, a mathematical model of the HIV pandemic can be reconstructed, and the disease's regulating systems may be studied. This theory explains how biological controls may be used to regulate epidemics and pandemics. Numerous researchers have adopted this idea about how to control infections. HIV models have been established recently to understand the behaviour of the virus after infection, HIV disease dynamics, the immune response, and the interactions of the virus with CD4 + T cells. Tripathi et al.^6^ presented an HIV mathematical model and claimed that HIV infection may be reduced significantly because of increased awareness of HIV-infected individuals identified by screening and contact tracing, but the illness remains prevalent due to immigration and the lack of contact tracing. They assessed that the most effective strategy for reducing the burden of the disease is to increase public awareness about HIV/AIDS. Nyabadza and Mukandavire^7^ investigated an HIV model that described HIV counselling and testing (HCT) and examined the influence of therapy during infection. Mushanyu^8^ suggested a model for HIV dynamics and examined the impact of delayed HIV diagnosis on the disease's emissions. They demonstrated HIV treatment motivation and enhanced HIV self-testing regimens that provide more undiagnosed individuals with the information necessary to determine their HIV status, ultimately minimising HIV transmission. Wang et al.^9^ explored the dynamics of an age-structured hybrid HIV/AIDS model with self-protection and media coverage. Granata et al.^10^ used an optimization technique to investigate the propagation of HIV-infected cells. Yuzbasi and Karacayir^11^ used the Galerkin scheme in order to solve the HIV transmission model. Attaullah and Sohaib^12^ employed Galerkin and Legendre wavelet collocation schemes for solving the HIV model. They also solved the model using the standard Runge–Kutta technique and compared the RK4-method findings to those obtained using the suggested techniques to validate their validity. Seatlhodi et al.^13^ proposed an entirely new HIV pandemic model that allows for an influx of new infected individuals into the community. They examined the impact of public health education initiatives on the prevalence of the condition and found that they had no effect. In order to define the control and determine the best system, they employed "Pontryagin's maximal principle." Arenas et al.^14^ concentrated on the mathematical analysis and numerical solution of a discrete time delay HIV model. Elaiw et al.^15^ developed an HTLV/HIV dual infection model. The model considers the role of the cytotoxic T lymphocyte (CTL) immune response in controlling the dual infection. The model demonstrates how uninfected CD4 + T cells interact with HIV-infected cells, HTLV-infected cells, free HIV particles, HIV-specific CTLs, and HTLV-specific CTLs. Parand et al.^16^ proposed an HIV model and solved it using the quasi-linearization-Lagrangian method. Ongun^17^ implemented the Laplace-Adomian decomposition method (LADM) to approximate the HIV infection solution. Merdan^18^ performed the variational iteration method (VIM) and modified VIM to provide an approximation of the HIV model. Yüzbaş^19^ used the Bessel collocation method to approximate the HIV infection solution. Doğan^20^ used the multistep LADM method for solving the HIV model. Gandomani^21^ applied the Müntz-Legendre polynomial approach and solved the HIV model. Several researchers in the literature presumed that HIV dynamics would occur with a stable supply of newly generated T cells from the thymus. However, instead of consistent occurrences, fluctuating phenomena have been observed due to the HIV infection's proclivity to infect these cells. We develop a new concept of numerous nonlinear variable source terms for thymic production of new T cells in order to depict more realistic behaviors. The computational analysis mentioned above has aroused our interest in implementing an innovative technique called the continuous Galerkin–Petrov scheme to determine an approximation to the nonlinear model. Investigate the effects of several variable source terms on the dynamics of the populations of healthy T-cells, infected T-cells, and free viruses. The model is critical for mathematically simulating HIV infection of T-cells. This will be used to analyse the population dynamics of T-cells in the presence and absence of HIV, which is beneficial for monitoring the clinically observed hallmarks of AIDS and slowing the disease's spread. This research will be a useful contribution to the existing body of information previously accessible on biomathematics. The following are the key contributions of the present investigation:

1. To implement the continuous Galerkin–Petrov time discretization scheme having polynomial order two for the novel HIV model including treatment rate.
2. To compare the solutions of the suggested methodology to the findings of the well-known Runge–Kutta method and other results obtained through conventional techniques exist in the literature.
3. To approximate numerical solutions with various time step sizes using the Runge–Kutta and Galerkin methods and to analyse the precision and validity of these approaches based on their absolute errors.
4. The fourth objective of the study is to improve a model by including treatment rates and analyses of the extended model based on the basic reproduction number and stability analysis.
5. To investigate the influence of different nonlinear and varied source terms for the growth of new healthy T-cells on the dynamical behaviour of the improved model.

## Mathematical model for HIV infection

In this section, a mathematical model for the HIV infection is considered. After HIV infection the blood is divided into three classes: uninfected class $T(t),$ infected class $I(t)$, and free HIV particles $V(t).$ The parameter $\gamma$ denotes population of uninfected T-cells, $\varpi$, $\zeta$ and $d$ represent death rate of uninfected and infected T-cells and virus respectively. The parameter $\rho$ shows the growth rate of uninfected T-cells, $M$ denotes the virus infection rate of uninfected T-cells, $\alpha$ shows virus particles per infected T-cells,${T}_{max}$ describes the maximum concentration of uninfected T-cells and $p$ shows the cure rate. The system of nonlinear differential equations proposed by Parand et al.^16^ is presented below:

1 $$\begin{aligned} \frac{dT}{{dt}} & = \gamma - \varpi T + \rho T\left( {1 - \frac{T + I}{{Tmax}}} \right) - MVT, \\ \frac{dI}{{dt}} & = MVT - \zeta I, \\ \frac{dV}{{dt}} & = \alpha \zeta I - dV, \\ \end{aligned}$$

The initial conditions for state variables are follows:

$$T \left(0\right)=0.1\mathrm{ m}{\mathrm{m}}^{-3} {\mathrm{day}}^{-1}, I \left(0\right)=0\mathrm{ m}{\mathrm{m}}^{-3}{ \mathrm{day}}^{-1}, V\left(0\right)=0.1\mathrm{ m}{\mathrm{m}}^{-3}{ \mathrm{day}}^{-1}.$$

**Figure 1:**
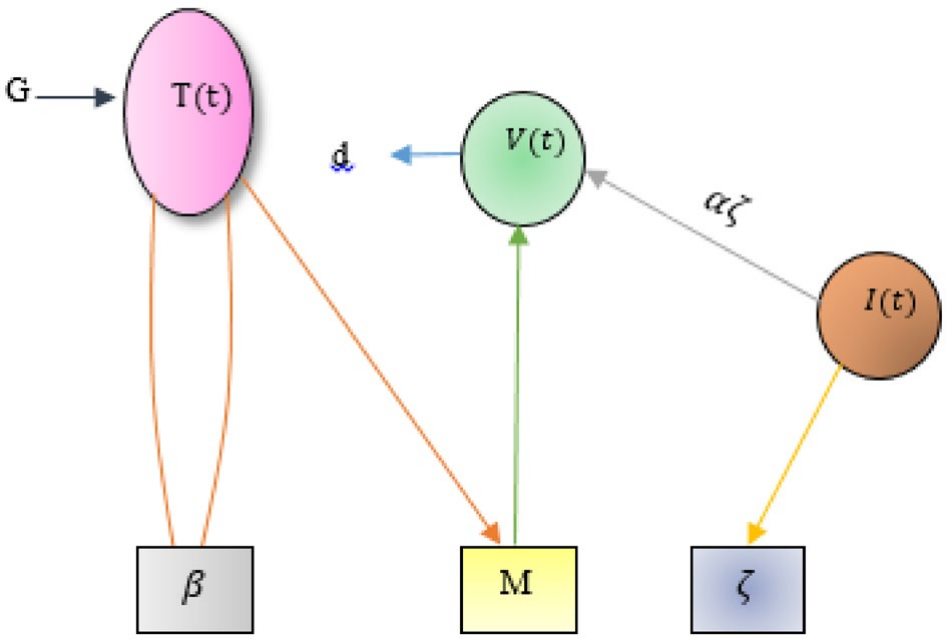
Diagrammatic illustration of the HIV infection model, where $G=\gamma +\rho T(1-\frac{T+I}{{T}_{max}})$.

The graphical illustration of the mathematical model of HIV infection is presented in Fig. 1. A comprehensive explanation of all the parameters involved in the model are summarized in Table 1.

**Table 1:** Explanation of the parameters involved in HIV infection model. The units are ${\mathrm{mm}}^{-3}{ day}^{-1}$. All parameter values are obtained from Parand et al.^16^.

Parameters	Explanation	Values
$\gamma$ $\varpi$ $\zeta$ $\rho$ $d$ $M$ $\alpha$ ${T}_{max}$ $p$	Population of uninfected T-cells Death rate of uninfected T-cells Death rate of infected T-cells Growth rate of T-cells Virus death rate Virus infection rate of T-cells Virus particles per infected T-cells Maximum population of healthy T-cells Rate of cure	0.1 $3$.0 0.3 $3$.0 2.4 0.0027 10 1500 0.01

## The continuous Galerkin Petrov technique

The Galerkin technique is an effective tool for numerically investigating critical challenges. This approach is commonly employed for complicated problems and is capable of dealing with nonlinear systems and complicated problems (see for detail^23–27^ information).

This section is focused with the application and implementation of the suggested technique to the model addressed by Parand et al.^16^. For simplicity some assumptions are given i.e., ${u}_{1}\left(t\right)=T\left(t\right) , { u}_{2}\left(t\right)=I\left(t\right), { u}_{3 }\left(t\right)=V\left(t\right),$ initially at $t=0,$

$$u_{1} \left( 0 \right) = T\left( 0 \right) = \rho_{1} , u_{2} \left( 0 \right) = I\left( 0 \right) = \rho_{2} , u_{3 } \left( 0 \right) = V\left( 0 \right) = \rho_{3} .$$

Find $u: \mathrm{J}=\left[0,T\right]\to V.$ Here $\mathrm{J}$ =$\left[0,T\right]$ describes the time interval, for function

2 $$\begin{gathered} {\varvec{u}}:{\text{J}} \times {\text{V}} \to {\text{and t}} \in T \hfill \\ d_{t} {\varvec{u}}\left( t \right) = \psi \left( {t,{\varvec{u}}\left( t \right)} \right)\forall t \in {\text{J}} = \left[ {0,T} \right]\user2{ u}\left( 0 \right) = {\varvec{u}}_{0} , \hfill \\ \end{gathered}$$

Here ${d}_{t}{\varvec{u}}\left(t\right)$ refers to the time derivative of ${\varvec{u}}\left(t\right)$. The derivative of $u\left(t\right)$ w.r.t. t ${\varvec{u}}\left(0\right)=$ (${u}_{1}\left(0\right),{u}_{2}\left(0\right),{u}_{3} (0))\in V$ represents ${\varvec{u}}$ (t) at *t* = 0, and $\psi$ $=({\psi }_{1},{\psi }_{2},{\psi }_{3})$ and described as $\psi :\mathrm{J}\times$ V $\to$ V. The formulation of Eq. (2) is: find ${\varvec{u}}\in {X}^{^{\prime}}$ such that ${\varvec{u}}\left(0\right)={{\varvec{u}}}_{{\varvec{o}}}$ and

3 $${\int }_{\mathrm{J}}\langle {d}_{t}{\varvec{u}}\left(t\right),\vartheta (t)\rangle dt={\int }_{\mathrm{J}}\langle \psi (\mathrm{t},{\varvec{u}}\left(t\right), \vartheta (t)\rangle dt \mathrm{for all }\vartheta \in {Y}^{^{\prime}},$$

where ${X}^{^{\prime}}$,and ${Y}^{^{\prime}}$ represent the solution space the test space respectively. To describe function t $\to$ ${\varvec{u}}\left(t\right)$, we consider the space $E$($\mathrm{J },\mathrm{V})={E}^{\mathrm{O}}$($\mathrm{J },\mathrm{V}$) as the space of contionuous functions ${\varvec{u}}:\mathrm{ J}\to$ V link up with norm

$${\varvec{u}}_{{E\left( {{\text{J }},{\text{V}}} \right) = {\text{E}}^{{\text{O}}} \left( {{\text{J }},{\text{ V}}} \right)}} = {\text{sup}}_{{{\text{t}} \in = \left[ {0,{\text{T}}} \right]}} {\varvec{u}}\left( t \right)V.$$

${\mathrm{M}}^{2}$($\mathrm{J },\mathrm{V})$ represents space of square intergrable function ${\mathrm{M}}^{2}$($\mathrm{J },\mathrm{V})$ by containing discontinuous functions, which is expressed in the form as ${\mathrm{M}}^{2}$($\mathrm{J },\mathrm{V})=\left\{{\varvec{u}}:\left[0,T\right]\to V:{\Vert {\varvec{u}}\Vert }_{{\mathrm{M}}^{2}(\mathrm{J },\mathrm{V}) }< \infty \right\}$ with ${\Vert {\varvec{u}}\Vert }_{{\mathrm{M}}^{2}(\mathrm{J },\mathrm{V}) }={ ({\int }_{\mathrm{J}}{\Vert {\varvec{u}}\left(t\right)\Vert V}^{2}dt)}^\frac{1}{2}$

We divide the time interval $J$ into $N$ subintervals for Galerkin time discretization.${J}_{n}=[{t}_{n-1} , {t}_{n}$], where $n=\mathrm{1,2},3,\dots N,$ and 0 = ${t}_{0}$<${t}_{1}$<$\dots {t}_{N-1}< {t}_{n}=T$. The symbol $\tau$ denotes the time discretization parameter, which will be used to determine the maximum time step size $\tau =\underset{1\le n\le N}{\mathrm{max}}{\tau }_{n}$, where ${\tau }_{n}={t}_{n}-{t}_{n-1}$, which is the length of each ${J}_{n}$. Now we will approximate ${\varvec{u}}:\mathrm{ J}\to \mathrm{V}$ using a function ${{\varvec{u}}}_{\tau }:\mathrm{J}\to \mathrm{V}$ (see^12, 22^ for details). Then, we will find the space for

$${{X}^{^{\prime}} }_{\tau }^{l}=\left\{{\varvec{u}}\in E\left(\mathrm{J}\to \mathrm{V}\right):{\varvec{u}}{|}_{{\mathrm{J}}_{n}}\in {H}_{l}\left({\mathrm{J}}_{n},V\right)\mathrm{for all }{\mathrm{J}}_{n}\in {G}_{\tau }\right\},$$

where $H_{l} \left( {{\text{J}}_{n} ,V} \right) = \left\{ {u:{\text{J}}_{n} \to {\text{V}},{\varvec{u}}\left( t \right) = \mathop \sum \limits_{{{\text{j}} = 0}}^{{\text{l}}} {\text{U}}^{{\text{j}}} {\text{t}}^{{\text{j}}} ,{ }\forall t \in {\text{J}}_{n} ,{\text{U}}^{{\text{j}}} \in V,for all j} \right\},$ and test space for ${{\varvec{u}}}_{\tau }$ is ${Y}_{\tau }^{k},$ illustrated as:

$${{Y}^{^{\prime}}}_{\tau }^{l}=\left\{V\in {\mathrm{M}}^{2}\left(\mathrm{J},\mathrm{V}\right):V{|}_{{\mathrm{J}}_{n}}\in {H}_{l-1}\left({\mathrm{J}}_{n},V\right)\mathrm{for all }{\mathrm{J}}_{n}\in {G}_{\tau }\right\},$$

where ${{Y}^{^{\prime}}}_{\tau }^{l}$ consists of piecewise polynomials of order $l-1$, which are discontinuous at the ends points of the time intervals. By taking a test function ${\vartheta }_{\tau }\in { {Y}^{^{\prime}}}_{\tau }^{l}$ and multiply it by Eq. (2), and integrate over $\mathrm{J}$ (see^12, 22^ for details). Find ${ {\varvec{u}}}_{\tau }\in { {X}^{^{\prime}}}_{\tau }^{l}$ such that ${{\varvec{u}}}_{\tau }\left(0\right)=0$ and

4 $$\mathop \smallint \limits_{{\text{J}}} d_{t} {\varvec{u}}_{\tau } \left( t \right),\vartheta_{\tau } \left( t \right)dt = \mathop \smallint \limits_{J} \psi \left( {{\text{t}},{\varvec{u}}_{\tau } \left( t \right)} \right), \vartheta_{{\tau { }}} \left( t \right)dt \forall \vartheta_{\tau } \in Y_{\tau }^{\prime l}$$

This discretization is called the exact cGP technique of order $l$ (see^12, 22^ for details). Now, to find $u{|}_{{\mathrm{J}}_{n}}\in {H}_{l}\left({\mathrm{J}}_{n},V\right)$ such that

5 $$\mathop \smallint \limits_{{{\text{J}}_{n} }} d_{t} {\varvec{u}}_{\tau } \left( t \right), \vartheta \varphi \left( {\text{t}} \right)dt = \mathop \smallint \limits_{{{\text{J}}_{n} }} \varphi \left( {{\text{t}},{\varvec{u}}_{\tau } \left( t \right)} \right),\vartheta \varphi \left( {\text{t}} \right)dt \forall \vartheta \in V and\forall { }\varphi \in H\left( {{\text{J}}_{n} } \right)$$

with the initial condition ${{\varvec{u}}}_{\tau {|}_{{\mathrm{J}}_{n}}}\left({t}_{n-1}\right)={{\varvec{u}}}_{{\varvec{\tau}}{|}_{{\mathrm{J}}_{n-1}}}\left({t}_{n-1}\right)$ for $n\ge 2$ and ${{\varvec{u}}}_{\tau {|}_{{\mathrm{J}}_{n}}}\left({t}_{n-1}\right)={{\varvec{u}}}_{0}$ for $n=1$. To find the integration on the right-hand side of Eq. (3.4), the $\left(l+1\right)$-points Gauss–Lobatto quadrature methodology will be used (see^12, 22^ for details). Find

6 $$\begin{aligned} & {\varvec{u}}|_{{{\text{J}}_{n} }} \in H_{l} \left( {{\text{J}}_{n} ,V} \right),{\text{ such that}} \\ & {\varvec{u}}_{\tau } \left( {t_{n - 1} } \right) = \user2{ u}_{n - 1} \\ & \mathop \sum \limits_{j = 0}^{l} w_{j} dt{\varvec{u}}_{\tau } \left( {t_{n,j} } \right)\varphi \left( {t_{n,j} } \right) = \mathop \sum \limits_{j = 0}^{l} w_{j} \psi \left( {{\text{t}}_{{{\text{n}},{\text{j}}}} , {\varvec{u}}_{\tau } \left( {t_{n,j} } \right)} \right){ }\varphi \left( {{\text{t}}_{{{\text{n}},{\text{j}}}} } \right)\forall { }\varphi \in H_{k - 1} ({\text{J}}_{n} ) \\ \end{aligned}$$

where ${w}_{j}$ are the weights.

To determine ${{\varvec{u}}}_{\tau }{|}_{{\mathrm{J}}_{n}}$, we represent it by a polynomial ansatz

7 $${\varvec{u}}_{\tau } \left( t \right) = \mathop \sum \limits_{j = 0}^{l} U_{n}^{j} \emptyset_{n,j} \left( {\text{t}} \right)\forall t \in {\text{J}}_{n}$$

where the coefficient ${U}_{n}^{j}$ is the elements of $V$ and the real valued function ${\varnothing }_{n,j}H\left({\mathrm{J}}_{n}\right)$ are the Lagrange basis functions with respect to $(l+1)$ suitable nodal points ${\mathrm{t}}_{\mathrm{n},\mathrm{j}}\in {\mathrm{J}}_{n}$ satisfying the conditions.

8 $${\varnothing }_{n,j}\left({t}_{n,j}\right)={\delta }_{i,j},i,j=\mathrm{0,1},2,\dots ,l,$$

where ${\delta }_{i,j}$ is the Kronecker delta that is,

$$\delta_{i,j} = \left\{ {\begin{array}{*{20}c} {1:} & {if i = j} \\ {0: } & {if i \ne j.} \\ \end{array} } \right.$$

For the choice of initial conditions, we set ${t}_{n,0}$=${t}_{n-1},$ which implies that the initial conditions for Eq. (5) is given as

9 $$\begin{aligned} U_{n}^{0} & = {\varvec{u}}_{\tau } |_{{{\text{J}}_{n} }} \left( {t_{n - 1} } \right),{\text{ if }}n \ge 2, \\ U_{n}^{0} & = {\varvec{u}}_{0} ,{\text{ if }}n = 1. \\ \end{aligned}$$

The other points ${t}_{n,1},{t}_{n,2},\dots \dots ,{t}_{tn,l}$ are selected as the $l$-points (quadrature points) of the Gauss–Lobatto formula on the interval ${\mathrm{J}}_{n}$. For representation (7), for ${d}_{t}{{\varvec{u}}}_{\tau }$, we get

10 $$d_{t} {\varvec{u}}_{\tau } = \mathop \sum \limits_{j = 0}^{l} U_{n}^{j} \emptyset^{\prime}_{n,j} \left( {\text{t}} \right),\forall {\text{t}} \in {\text{J}}_{n} ,$$

Using Eq. (10) in Eq. (5), we get

$${\int }_{{\mathrm{J}}_{n}}\langle {d}_{t}{{\varvec{u}}}_{\tau }\left(t\right), \vartheta \rangle \mathrm{\varphi }\left(\mathrm{t}\right)dt={\int }_{{\mathrm{J}}_{n}}\langle \sum_{j=0}^{l}{ U}_{n}^{j},\vartheta \rangle {{\varnothing }^{^{\prime}}}_{j}(\mathrm{t})\mathrm{ \varphi }\left(\mathrm{t}\right)dt.$$

This implies that

11 $${\int }_{{\mathrm{J}}_{n}}\langle {d}_{t}{{\varvec{u}}}_{\tau }\left(t\right), \vartheta \rangle \mathrm{\varphi }\left(\mathrm{t}\right)dt=\sum_{j=0}^{l}\langle { U}_{n}^{j},\vartheta \rangle {\int }_{{\mathrm{J}}_{n}}{{\varnothing }^{^{\prime}}}_{j}(\mathrm{t})\mathrm{ \varphi }\left(\mathrm{t}\right)dt$$

we define the basis functions ${\varnothing }_{n,j}{\in H}_{k}\left({\mathrm{J}}_{n}\right)$ via the affine reference transformation${\varpi }_{n}$: ${\hat{\mathrm{j}} }\to {\mathrm{J}}_{n},$ where ${\hat{\mathrm{j}} }=[-\mathrm{1,1}]$ and

12 $$t = \varpi_{n} \hat{t} = \frac{{t_{n} + t_{n - 1} }}{2} + \frac{{\tau_{n} }}{2}\hat{t} \in J_{n} \forall \hat{t} \in \hat{j},n = 1,2,3, \ldots ,N.$$

Let $\widehat{\varnothing }$ j $\in$ ${H}_{k}({ \hat{\mathrm j} })$, $j=\mathrm{0,1},2,\dots ,l,$ denote the basis functions satisfying the conditions

13 $$\widehat{\varnothing }({\widehat{t}}_{i})= {\delta }_{i,j} , i,j=\mathrm{0,1},2,\dots ,l,$$

Then, we define the basis functions on the original time interval ${\mathrm{J}}_{n}$ by the mapping (see^12, 22^ for details as follows:

$${\varnothing }_{n,j}\left(t\right)=\widehat{\varnothing }\mathrm{j }\left(\widehat{t}\right)\mathrm{with }\widehat{t}={ \varpi }_{n}^{-1}\left(t\right)=\frac{2}{{\tau }_{n}}\left(t+\frac{{t}_{n-1-{t}_{n}}}{2}\right)\in {\hat{\mathrm{j}} }.$$

Furthermore, we provide the test basis functions ${\mathrm{\varphi }}_{n,i}$ by using appropriate reference basis functions

14 $$\begin{gathered} \hat{\varphi }_{i} \in H_{l - 1} \left( {\hat{j}} \right),i.e, \hfill \\ \varphi_{n,i} \left( t \right) = \hat{\varphi }_{i} ( \varpi_{n}^{ - 1} \left( t \right) \quad \forall t \in J_{n} , i = 1,2,3, \ldots ,l. \hfill \\ \end{gathered}$$

Now, we transform the integration into a reference interval ${\hat{\mathrm{j}} }=[-\mathrm{1,1}]$ and $(l+1)$ point Gauss–Lobatto quadrature technique is used to approximate it for each test basis function $\mathrm{\varphi }\in {H}_{l-1}$ and for all $\vartheta \in V$ as follows:

$$\mathop \smallint \limits_{{ \hat{j}_{n} }} \mathop \sum \limits_{j = 0}^{l} U_{n}^{j} ,\vartheta \hat{\emptyset }_{j}^{^{\prime}} \left( {\hat{t}} \right) \hat{\varphi }\left( {\hat{t}} \right)d_{{\hat{t}}} = \frac{{\tau_{n} }}{2} \mathop \smallint \limits_{{ \hat{j}_{n} }} \psi \left( {\omega_{n} \left( {\hat{t}} \right),\mathop \sum \limits_{j = 0}^{l} U_{n}^{j} \left( {\hat{t}} \right)} \right),\vartheta \hat{\varphi }\left( {\hat{t}} \right)d_{{\hat{t}}} \;\forall \vartheta \in V$$

This implies that

15 $$\sum_{\mu =0}^{l}\widehat{{ \varpi }_{\mu }}\sum_{j=0}^{l}\langle { U}_{n}^{j},\vartheta \rangle {\widehat{\varnothing }}_{j}^{^{\prime}}\left({\widehat{t}}_{\mu }\right)\widehat{\mathrm{\varphi }}\left({\widehat{t}}_{\mu }\right)=\frac{{\tau }_{n}}{2}\sum_{\mu =0}^{l}\widehat{{ \varpi }_{\mu }}\langle \psi \left({\upomega }_{\mathrm{n}}{\widehat{t}}_{\mu },\sum_{\mathrm{j}=0}^{\mathrm{l}}{ U}_{n}^{j}\left({\widehat{t}}_{\mu }\right)\right),\vartheta \rangle \widehat{\mathrm{\varphi }}\left({\widehat{t}}_{\mu }\right).$$

Here $\widehat{{\varpi }_{\mu }}$ are the weights and ${\widehat{t}}_{\mu }$ $\in$ [1, −1] are the integration points with the ${\widehat{t}}_{0}=-1$ and ${\widehat{t}}_{l}=1$

16 $${\widehat{\mathrm{\varphi }}}_{i}\left({\widehat{t}}_{\mu }\right)={(\widehat{{ \varpi }_{\mu }})}^{-1}{\delta }_{i,\mu } i,\mu =\left\{\mathrm{1,2},3,\dots ,l\right\},$$

Afterwards, find the $l$ unknown coefficients ${U}_{n}^{j}\in V$ where $j=\mathrm{1,2},3,\dots ,l,$ such that

17 $$\mathop \sum \limits_{j = o}^{l} \hat{\varpi }_{i,j} U_{n}^{j} = \frac{{\tau_{n} }}{2}\left\{ {\psi \left( {{\text{t}}_{{{\text{n}},{\text{i}}}} ,U_{n}^{j} } \right) + \sigma_{i} { }\psi \left( {{\text{t}}_{{{\text{n}},0}} ,U_{n}^{o} } \right)} \right\} \forall i = 1,2,3, \ldots l$$

where ${U}_{n}^{o}={ U}_{n-1}^{l} for n>1 and { U}_{1}^{o}={{\varvec{u}}}_{0\boldsymbol{ }}for n=1,$ indicated intial values and ${z}_{i,j}$ and ${\sigma }_{i}$ are define as

18 $${\widehat{\varpi }}_{i,j}={\widehat{\varnothing }}_{j}^{^{\prime}}\left({\widehat{t}}_{\mu }\right)+{\sigma }_{i}{\widehat{\varnothing }}_{j}^{^{\prime}}\left({\widehat{t}}_{\mu }\right),{t}_{n,\mu }={\widehat{\varpi }}_{n}\left({\widehat{t}}_{\mu }\right)\mathrm{and}{ \sigma }_{i}={\widehat{\varpi }}_{0}{\widehat{\mathrm{\varphi }}}_{i}\left({\widehat{t}}_{\mu }\right).$$

### The cGP(2)-scheme

Here, Gauss–Lobatto formula along the points ${t}_{n,0}={t}_{n-1}$, ${t}_{n,1}=\left(\frac{{t}_{n}+{t}_{n-1}}{2}\right), {t}_{n,2}={t}_{n}$ and the weights $\widehat{{\varpi }_{0}}=\widehat{{ \varpi }_{2}}=\frac{1 }{3}$, $\widehat{{\varpi }_{1}}=\frac{4}{3}$ are used to get the coefficients

$${\widehat{\varpi }}_{i,j}=\left(\begin{array}{ccc}\frac{-5}{4}& 1& \frac{1}{4}\\ 2& -4& 2\end{array}\right),{ \sigma }_{i}=\left(\begin{array}{c}\frac{1}{2}\\ -1\end{array}\right),i=\mathrm{1,2} j=\mathrm{0,1},2.$$

with respect to the time interval ${{\hat{\mathrm{j}} }}_{n}=\left]{t}_{n-1},{t}_{n}\right]$, The system can be determined for two unknowns such as

${U}_{n}^{j}={{\varvec{u}}}_{{\varvec{\tau}}}({t}_{n,j})$ with ${t}_{n,j}={\varpi }_{n}(\widehat{t})$ for $j=(\mathrm{1,2}$). The couple (2 × 2) block-system for ${U}_{n}^{1}{ ,U}_{n}^{2} \in V$, is as follows:

19 $${\widehat{\varpi }}_{\mathrm{1,1}}{U}_{n}^{1}+{\widehat{\varpi }}_{\mathrm{1,2}}{U}_{n}^{2}=-{\widehat{\varpi }}_{\mathrm{1,0}}{ U}_{n}^{0}+\frac{{\tau }_{n}}{2}\left\{\psi \left({\mathrm{t}}_{\mathrm{n},1 },{U}_{n}^{1}\right)+{\sigma }_{1}\psi \left({\mathrm{t}}_{\mathrm{n},0 },{U}_{n}^{o}\right)\right\},$$

20 $${\widehat{\varpi }}_{\mathrm{2,1}}{U}_{n}^{1} +{\widehat{\varpi }}_{\mathrm{2,2}}{U}_{n}^{2}=-{\widehat{\varpi }}_{\mathrm{2,0}}{ U}_{n}^{0}+\frac{{\tau }_{n}}{2}\left\{\psi \left({\mathrm{t}}_{\mathrm{n},2 },{U}_{n}^{2}\right)+{\sigma }_{2}\psi \left({\mathrm{t}}_{\mathrm{n},0 },{U}_{n}^{o}\right)\right\},$$

${U}_{n}^{0}$ indicates the initial value at the time interval ${{\hat{\mathrm{j}} }}_{n}$ obtaining from the time interval or the intial value ${{\varvec{u}}}_{0}$.

### The Runge–Kutta scheme

This well-known scheme is established by Kutta having order four (see^37^ for details information).

## Comparative analysis of present scheme with other conventional schemes

**Figure 2:**
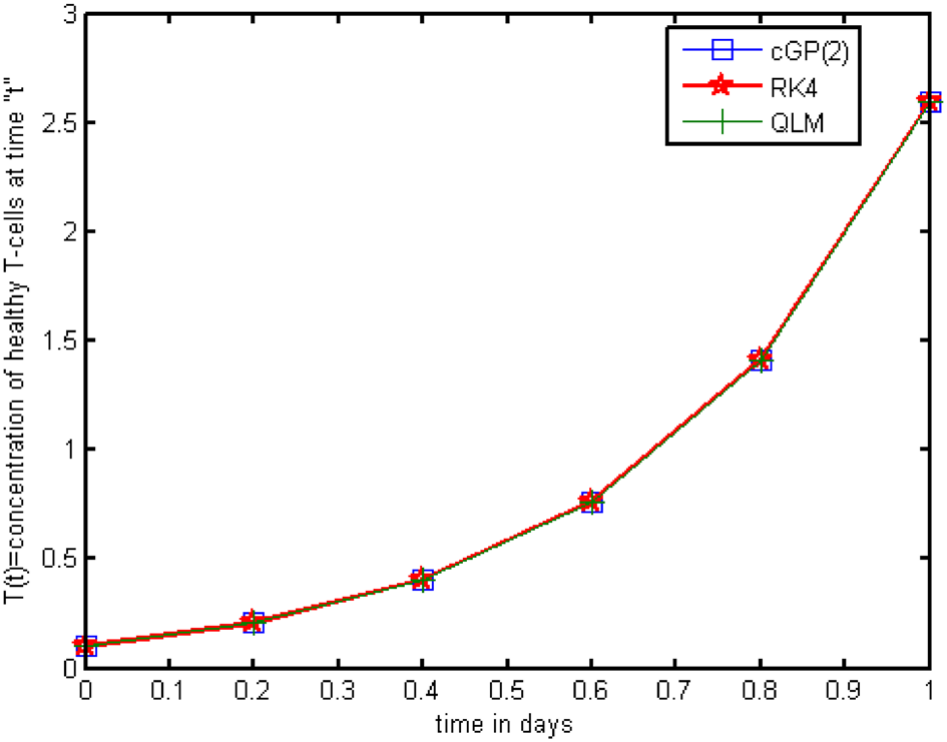
Graphical comparison of absolute errors for concentration of healthy T-cells.

**Figure 3:**
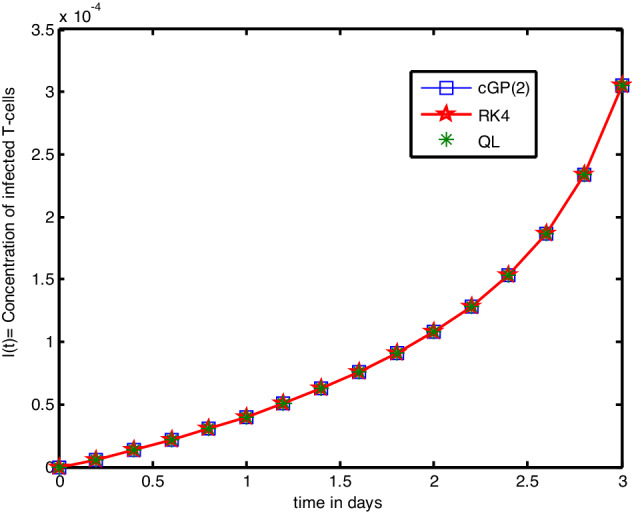
Graphical comparison of absolute errors for concentration of infected T-cells.

**Figure 4:**
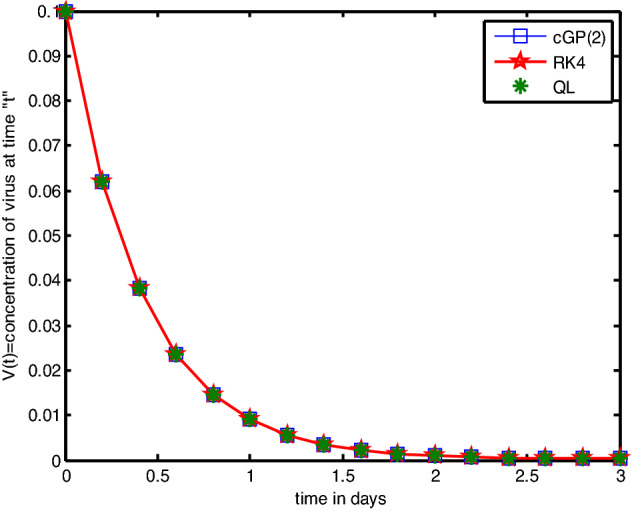
Graphical comparison of absolute errors for virus.

**Figure 5:**
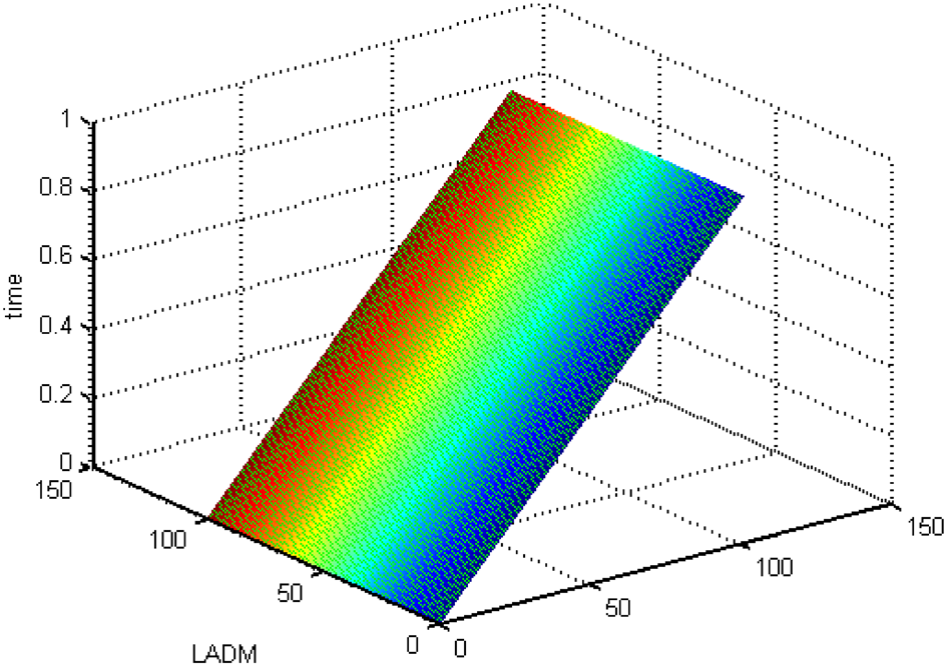
The mesh grid graph for LADM.

**Figure 6:**
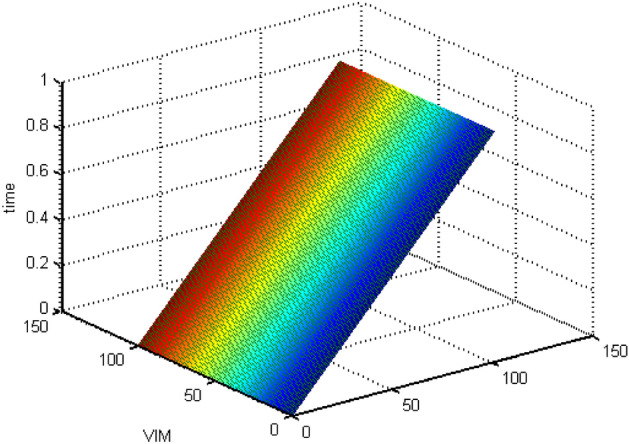
The mesh grid graph for VIM.

**Figure 7:**
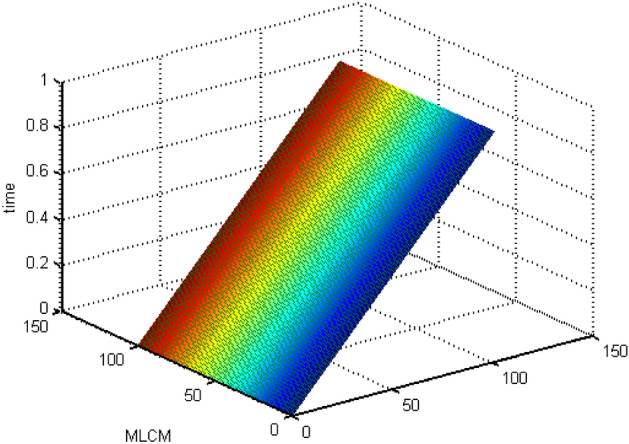
The mesh grid graph for MLCM.

**Figure 8:**
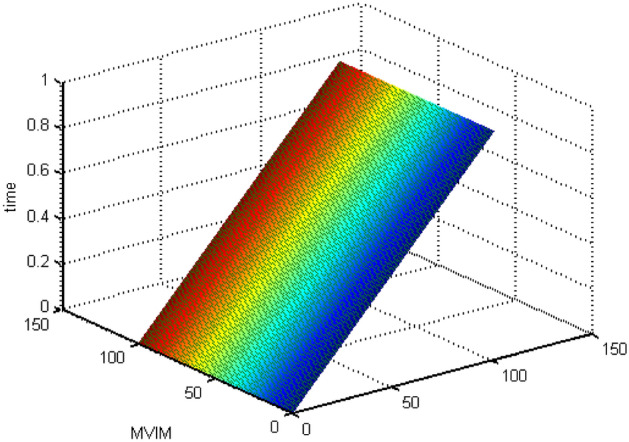
The mesh grid graph for MVIM.

**Figure 9:**
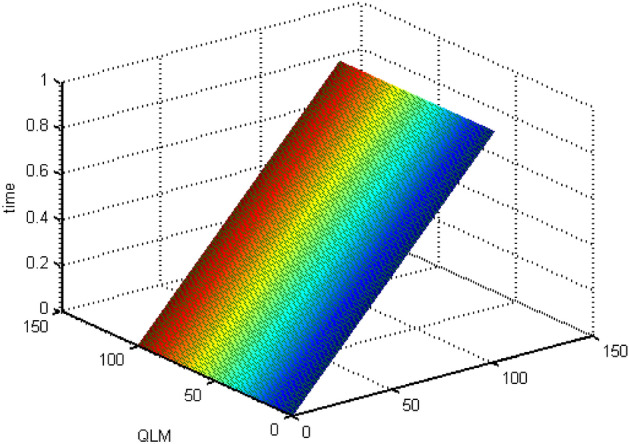
The mesh grid graph for QLM.

In this section, we implemented the Galerkin and RK4 techniques and compared the outcomes with those achieved through other conventional techniques. Tables 2, 3, 4, 5, 6, 7, 8, 9, 10, 11, 12, 13, 14, 15 present the numerical results, whereas Figs. 2, 3, 4 show the graphical results, and the absolute errors between the outputs of the Galerkin and Q-L methods were analysed in comparison to the results of the traditional RK4 technique. The findings obtained by the Galerkin and RK4 approaches that overlap each other throughout the entire domain demonstrate the reliability and validity of the implemented techniques. Tables 2, 3, 3 indicated that the errors are extremely small and the results are very close to each other, which assures the accuracy of the suggested schemes and codes. We also compared the RK4 and Galerkin methods' results to those obtained by other conventional techniques, such as LADM^17^, VLM^18^, MLCM^20^, and MVIM^18^, as shown in Tables 5, 6, 7 for and, respectively. A comparative assessment revealed vividly that the mentioned methods yield more accurate findings. Furthermore, we investigated the outputs and absolute errors relative to the RK4 method for and of the proposed schemes with traditional techniques, as shown in Tables 8, 9, 10. It demonstrated that our approximate solutions are more comparable to RK4-method solutions than the solutions obtained via previously developed methods. Afterwards, we performed different numerical experiments for the proposed scheme and the RK4 scheme with the same and different step sizes shown in Tables 11, 12, 13, 14, 15. It revealed that Galerkin solutions with larger step sizes obtained significant accuracy as compared to RK4 solutions with much smaller time steps. Furthermore, the results of the Galerkin, RK4, and Q-L methods are depicted graphically in Figs. 2, 3, 4. It has been observed that the suggested method performs well for finding solutions to real-world problems. Additionally, we presented the mesh grid graphs in Figs. 5, 6, 7, 8, 9 for the results of all the methods used for the above model.

**Table 2:** Comparative analysis for *T(t)* with RK4, |QLM-RK4| and |Galerkin-RK4|.

*t*	Galerkin	Q-L method^1^	RK4-method	|QLM-RK4|	|Galerkin-RK4|
0.0	0.1000000000000	0.1000000000000	0.1000000000000	0.000000000000000	0.000000000000000
0.2	0.2088079854767	0.2088080843259	0.2088075606565	0.000000523669510	0.000000424820214
0.4	0.4062401842998	0.4062405427886	0.4062386428473	0.000001899941428	0.000001541452568
0.6	0.7644229239703	0.7644238985049	0.7644187304208	0.000005168084188	0.000004193549580
0.8	1.4140444992445	1.4140468518885	1.4140343636464	0.000012488242188	0.000010135598083
1.0	2.5915895355574	2.5915948518626	2.5915665907902	0.000028261072428	0.000022944767145
1.2	4.7239535650329	4.7239650647145	4.7239037826421	0.000061282072517	0.000049782390844
1.4	8.5783869462398	8.5784110217119	8.5782822405967	0.000128781115292	0.000104705643135
1.6	15.522907829643	15.522956803180	15.522693209630	0.000263593549379	0.000214620012294
1.8	27.961562951032	27.961659661690	27.961133839532	0.000525822158401	0.000429111500811
2.0	50.008412764626	50.008597034795	50.007578859332	0.001018175463273	0.000833905294527
2.2	88.367937884312	88.368272307407	88.366377456808	0.001894850599157	0.001560427503478
2.4	53.006888073211	153.00745446872	53.004124842076	0.003329626644501	0.002763231134622
2.6	56.267306528950	256.26817818681	56.262801069901	0.005377116909926	0.004505459049255
2.8	407.94071286853	407.94189926565	407.93419138481	0.007707880839178	0.006521483718359
3.0	605.33064968016	605.33204616786	605.32257582540	0.009470342465988	0.008073854766621

**Table 3:** Comparative analysis for *I(t)* with RK4, |QLM-RK4| and |Galerkin-RK4|.

*t*	Galerkin	Q-L scheme	RK4-scheme	|QLM-RK4|		|Galerkin-RK4|
0.0	0.000000E−0	0.00000000E−0	0.000000E−0		0.0000E−00	0.0000E−00
0.2	0.603269E−5	0.60327022E−5	0.603264E−5		5.3682E−11	4.3995E−11
0.4	0.131583E−4	0.13158340E−4	0.131582E−4		1.2320E−10	1.0067E−10
0.6	0.212237E−4	0.21223785E−4	0.212235E−4		2.1007E−10	1.7116E−10
0.8	0.301773E−4	0.30177420E−4	0.301771E−4		3.1673E−10	2.5739E−10
1.0	0.400377E−4	0.40037815E−4	0.400373E−4		4.4638E−10	3.6195E−10
1.2	0.508784E−4	0.50878544E−4	0.508779E−4		6.0306E−10	4.8811E−10
1.4	0.628271E−4	0.62827260E−4	0.628264E−4		7.9177E−10	6.4005E−10
1.6	0.760822E−4	0.76082488E−4	0.760814E−4		1.0191E−10	8.2342E−10
1.8	0.909592E−4	0.90959537E−4	0.909582E−4		1.2947E−09	1.0466E−10
2.0	0.107990E−3	0.10799080E−3	0.107989E−3		1.6341E−09	1.3238E−10
2.2	0.128129E−3	0.12812960E−3	0.128127E−3		2.0656E−09	1.6802E−10
2.4	0.153143E−3	0.15314397E−3	0.153141E−3		2.6422E−09	2.1636E−10
2.6	0.186329E−3	0.18633049E−3	0.186327E−3		3.4619E−09	2.8616E−10
2.8	0.233693E−3	0.23369467E−3	0.233690E−3		4.6562E−09	3.9285E−10
3.0	0.305684E−3	0.30568418E−3	0.305678E−3		5.7410E−09	5.6223E−10

**Table 4:** Comparative analysis for *V(t)* with RK4, |QLM-RK4| and |Galerkin-RK4|.

*t*	Galerkin	Q-L scheme	RK4-scheme		|QLM-RK4|	|Galerkin-RK4|
0.0	1.000000000E−1	1.000000000000E−1	1.000000000E−1		0.00000E−00	0.0000E−00
0.2	6.187985172E−2	6.18798432237E−2	6.187989993E−2		5.67597E−08	4.8200E−08
0.4	3.829489839E−2	3.82948877731E−2	3.829495805E−2		7.02324E−08	5.9646E−08
0.6	2.370455988E−2	2.37045500445E−2	2.370461520E−2		6.51562E−08	5.5342E−08
0.8	1.468037171E−2	1.46803636840E−2	1.468041739E−2		5.36950E−08	4.5617E−08
1.0	9.100851256E−3	9.10084499664E−3	9.100886468E−3		4.14301E−08	3.5211E−08
1.2	0.565328299E−2	0.56532784219E−2	0.565330903E−2		3.06099E−08	2.6032E−09
1.4	0.352542932E−2	0.35254260732E−2	0.352544795E−2		2.18791E−08	1.8630E−09
1.6	0.221476879E−2	0.22147665690E−2	0.221478174E−2		1.51729E−09	1.2948E−09
1.8	0.141047146E−2	0.14104700109E−2	0.141048017E−2		1.01619E−09	8.7084E−09
2.0	0.920401419E−3	0.92040054118E−3	0.920407000E−3		6.45908E−09	5.5804E−09
2.2	0.626019016E−3	0.62601856977E−3	0.626022273E−3		3.70384E−09	3.2576E−09
2.4	0.454616816E−3	0.45461670237E−3	0.454618288E−3		1.58599E−09	1.4721E−09
2.6	0.362403747E−3	0.36240386282E−3	0.362403731E−3		1.30885E−10	1.5827E−11
2.8	0.324492781E−3	0.32449257921E−3	0.324491335E−3		1.24344E−09	1.4452E−09
3.0	0.329409100E−3	0.32940341266E−3	0.329406006E−3		2.59412E−09	3.0936E−09

**Table 5:** Comparative analysis of the findings of Galerkin scheme and other classical schemes for *T(t).*

*t*	Galerkin	RK4-method	LADM^17^	VIM^18^	MLCM^20^	MVIM^18^
0.0	0.100000000	0.100000000	0.100000000	0.100000000	0.10000000	0.100000000
0.2	0.208806496	0.208800678	0.208807329	0.208807321	0.2088080840	0.208808086
0.4	0.406234784	0.406213674	0.406135831	0.406134658	0.406240543	0.406240794
0.6	0.764408254	0.764350814	0.762476222	0.762453035	0.766442390	0.764428724
0.8	1.414009061	1.413870248	1.398082863	1.397880588	1.414046852	1.414094173
1.0	2.591509458	2.591195190	2.507874151	2.506746669	2.591559480	2.591921076

**Table 6:** Comparative analysis of the findings of Galerkin scheme and other classical schemes for *I(t).*

*t*	Galerkin	RK4 method	LADM^17^	VIM^18^	MLCM^20^	MVIM^18^
0.0	0.000000000E−0	0.000000000E−0	0.00000000E−0	0.0000000E−0	0.000000E−0	0.10000000E−13
0.2	6.032546494E−6	6.031878972E−6	0.60327069E−5	0.6032634E−5	0.603270E−5	0.60327016E−05
0.4	1.315797854E−5	1.315648606E−5	0.13158910E−4	0.1314878E−4	0.131583E−4	0.13158301E−04
0.6	2.122315973E−5	2.122067760E−5	0.21232981E−4	0.2101417E−4	0.212237E−4	0.21223310E−04
0.8	3.017646616E−5	3.017281029E−5	0.30242701E−4	0.2795130E−4	0.301774E−4	0.30174509E−04
1.0	4.003645811E−5	4.003141584E−5	0.40333218E−4	0.2431562E−4	0.400378E−4	0.40025404E−04

**Table 7:** Comparative analysis of the findings of Galerkin scheme and other classical schemes for *V(t).*

*t*	Galerkin	RK4-method	LADM^17^	VIM^18^	MLCM^20^	MVIM^18^
0.0	0.10000000000	0.10000000000	0.10000000000	0.10000000000	0.100000000	0.1000000000
0.2	0.06187998051	0.06188084740	0.06187995305	0.06187995314	0.061879843	0.0618799087
0.4	0.03829505758	0.03829613043	0.03830818047	0.03830820126	0.038294888	0.0382959576
0.6	0.02370470746	0.02370570312	0.02391981608	0.02392029257	0.023704550	0.0237102948
0.8	0.01468049325	0.01468131432	0.01621234343	0.01621704553	0.014680364	0.0147004190
1.0	0.00910094475	0.00910157907	0.01605502238	0.01608418711	0.009100845	0.0091572387

**Table 8:** Comparative analysis of absolute errors between Galerkin scheme and conventional approaches relative to RK4 technique for $T(t).$

*t*	0.1	0.2	0.4	0.6	0.8	1.0
cGP(2)	0.0	5.18760E−6	2.11093E−5	5.74299E−5	1.38812E−4	3.14268E−4
LADM^17^	0.0	6.65092E−6	7.78434E−5	1.87459E−3	1.57873E−2	8.33210E−2
VIM^18^	0.0	6.64252E−6	7.90162E−5	1.89777E−3	1.59896E−2	8.44485E−2
MLCM^20^	0.0	7.40512E−6	2.68680E−5	2.09157E−3	1.76603E−4	3.64289E−4
MVIM^18^	0.0	7.40792E−3	2.71193E−2	7.77909E−2	2.23924E−1	7.25885E−1

**Table 9:** Comparative analysis of absolute errors between Galerkin scheme and conventional approaches relative to RK4 technique for $I(t).$

*t*	0.1	0.2	0.4	0.6	0.8	1.0
cGP(2)	0.0	6.67521E−10	1.49248E−9	2.48213E−9	3.65587E−9	5.04227E−9
LADM^17^	0.0	8.27884E−10	2.42395E−9	1.23041E−8	6.98912E−8	3.01802E−7
VIM^18^	0.0	7.55394E−10	7.77006E−9	2.06505E−7	2.22150E−6	1.57157E−5
MLCM^20^	0.0	8.23268E−10	1.85483E−9	3.10779E−9	4.60980E−9	6.39965E−9
MVIM^4^	0.0	8.22679E−07	1.81560E−6	2.63240E−6	1.69902E−6	6.01179E−6

**Table 10:** Comparative analysis of absolute errors between Galerkin scheme and conventional approaches relative to RK4 technique for $V(t).$

*t*	0.1	0.2	0.4	0.6	0.8	1.0
cGP(2)	0.0	8.66885E−7	1.07285E−6	9.95660E−7	8.21068E−7	6.34323E−7
LADM^17^	0.0	8.94351E−7	1.20500E−5	2.14112E−4	1.53102E−3	6.95344E−3
VIM^18^	0.0	8.94261E−7	1.20708E−5	2.14112E−4	1.53573E−3	6.98260E−3
MLCM^20^	0.0	1.00440E−6	1.24243E−6	2.14112E−4	9.50322E−7	7.34076E−7
MVIM^18^	0.0	9.38641E−4	1.72757E−4	4.59167E−3	1.91046E−2	7.34076E−7

**Table 11:** Analysis of absolute errors between Galerkin and RK4 results for the similar step sizes.

		|cGP(2)τ = 0.1-RK4τ = 0.1|	
*t*	*T(t)*	*I(t)*	*V(t)*
0.0	0.0000000E−0	0.0000000E−00	0.0000000E−0
0.2	5.8176074E−6	6.6752196E−10	8.6688500E−7
0.4	2.1109347E−5	1.4924805E−09	1.0728542E−6
0.6	5.7429902E−5	2.4821350E−09	9.9566078E−7
0.8	1.3881221E−4	3.6558718E−09	8.2106806E−7
1.0	3.1426857E−4	5.0422736E−09	6.3432370E−7

**Table 12:** Comparison of the absolute errors of Galerkin scheme and RK4 for *T(t).*

*t*	cGP(2)τ = 0.2	RK4τ = 0.01	|cGP(2)-RK4|
0.0	1.000000E−1	1.0000E−1	0.000000E−0
0.2	2.087822E−1	2.0880E−1	2.580348E−5
0.4	4.061469E−1	4.0624E−1	9.357408E−5
0.6	7.641695E−1	7.6442E−1	2.543614E−4
0.8	4.134328E−0	1.4140E−0	6.140232E−4
1.0	2.590207E−0	2.5910E−0	1.387372E−3

**Table 13:** Absolute errors between the findings of Galerkin scheme and RK4 for *I(t).*

*t*	cGP(2)τ = 0.2	RK4τ = 0.01	|Galerkin-RK4|
0.0	0.0000E−0	0.0000E−0	0.000000E−0
0.2	6.0301E−6	6.0327E−6	2.539618E−9
0.4	1.3152E−5	1.3158E−5	5.907922E−9
0.6	2.1213E−5	2.1223E−5	1.019899E−9
0.8	3.0161E−5	3.0177E−5	1.554771E−8
1.0	4.0015E−5	4.0037E−5	2.212079E−8

**Table 14:** Absolute errors between the findings of Galerkin scheme and RK4 for *V(t).*

*t*	cGP(2)τ = 0.2	RK4τ = 0.01	|cGP(2)-RK4|
0.0	1.000000E−1	1.000000E−1	0.000000E−0
0.2	6.188206E−2	6.187984E−2	2.219134E−6
0.4	3.829763E−2	3.829488E−2	2.744747E−6
0.6	2.370709E−2	2.370455E−2	2.544546E−6
0.8	1.468245E−2	1.468036E−2	2.094335E−6
1.0	9.102457E−3	9.100845E−3	1.612383E−6

**Table 15:** Absolute errors between the findings of Galerkin scheme and RK4 with different step sizes.

		|cGP(2)τ = 0.2-RK4τ = 0.01|	
*t*	*T(t)*	*I(t)*	*V(t)*
0.0	0.000000E−0	0.000000E−0	0.000000000E−0
0.2	2.580348E−5	2.539618E−9	2.219134007E−6
0.4	9.357408E−5	5.907922E−9	2.744747372E−6
0.6	2.543614E−4	1.019899E−9	2.544546229E−6
0.8	6.140232E−4	1.554771E−8	2.094335180E−6
1.0	1.387372E−3	2.212079E−8	1.612383063E−6

## Mathematical formulation of modified model for HIV infection

**Figure 10:**
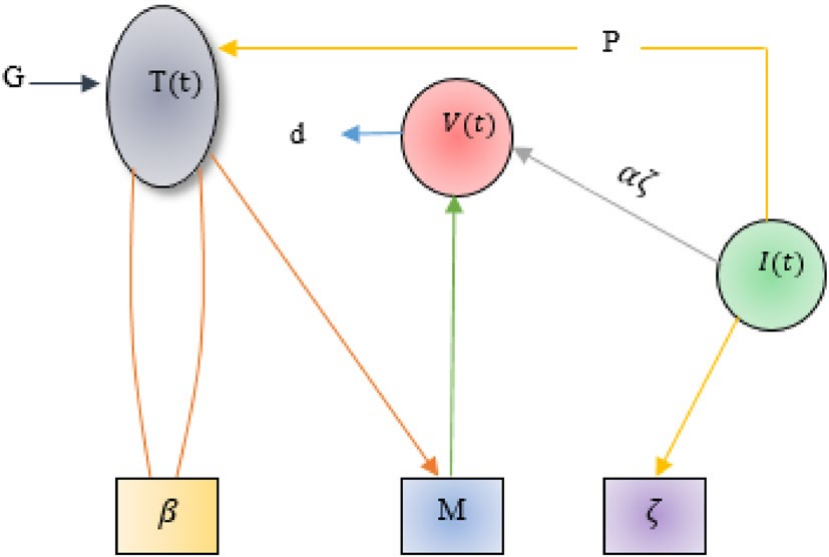
Diagrammatic representation of the mathematical Model (21) for HIV infection.

This section presents a mathematical model describing the population dynamics of healthy T cells, infected T cells, and the HIV virus. This model is an enhancement of a previously examined model introduced by Parand et al.^16^ by introducing the cure rate. The model is compartmentalised into three classes described as follows:

21 $$\begin{gathered} \frac{dT}{{dt}} = \gamma - \varpi T + \rho T\left( {1 - \frac{T + I}{{Tmax}}} \right) - MVT + pI, \hfill \\ \frac{dI}{{dt}} = MVT - \zeta I - pI, \hfill \\ \frac{dV}{{dt}} = \alpha \zeta I - dV. \hfill \\ \end{gathered}$$

where $T$, $I$ and $V$ represent the concentration of uninfected T-cells, infected T-cells and free virus particle respectively. The detail explanation of all parameters are presented in Table 1. The pictorial representation of the mathematical model (21) of HIV infection visualized in Fig. 10.

The initial conditions are follows:

$$T \left(0\right)=0.1\mathrm{ m}{\mathrm{m}}^{-3}{ \mathrm{day}}^{-1}, I \left(0\right)=0\mathrm{ m}{\mathrm{m}}^{-3}{ \mathrm{day}}^{-1}, V\left(0\right)=0.1\mathrm{ m}{\mathrm{m}}^{-3}{ \mathrm{day}}^{-1}.$$

### Basic reproduction number (${R}_{0}$)

The basic reproduction number is used to examine disease transmission, depicts the increase and controlling of the illness. If ${\mathrm{R}}_{0}<1$ then the disease-free equilibrium is stable, and the disease stops existing in the community. If ${\mathrm{R}}_{0}>1$, the endemic equilibrium exists because the disease spreads throughout the community. The reproduction number is obtained using the Next-generation matrix.

Let $X = (I, V),$ then, based on Model (21):

$$\frac{\mathrm{dX}}{\mathrm{dt}}= \mathcal{F}-\mathcal{V},$$

$$\mathcal{F}=\left(\begin{array}{c}\mathrm{MVT}\\ 0\end{array}\right)\mathrm{ and }\,\,\mathcal{V}=\left(\begin{array}{c}\left(\upzeta +\mathrm{p}\right)\mathrm{I}\\ -\mathrm{\alpha \zeta I}+\mathrm{dV}\end{array}\right).$$

The Jacobian matrix of $\mathcal{F}\mathrm{ and }\mathcal{V}$ are as follows:

$$\mathcal{F}=\left(\begin{array}{cc}0& \mathrm{MT}\\ 0& 0\end{array}\right)\mathrm{ and }\,\,\mathcal{V}=\left(\begin{array}{cc}(\upzeta +\mathrm{p})& 0\\ -\mathrm{\alpha \zeta }& \mathrm{d}\end{array}\right).$$

The next-generation matrix for the System (21) is

$$\mathcal{F}{\mathcal{V}}^{-1}=\left(\begin{array}{cc}\frac{\mathrm{M\alpha \zeta T}}{\left(\upzeta +\mathrm{p}\right)\mathrm{d}}& \frac{\mathrm{M}}{\mathrm{d}}\\ 0& 0\end{array}\right).$$

The eigenvalues of the matrix $\mathcal{F}{\mathcal{V}}^{-1}$ is ${\uplambda }_{1}=0\mathrm{ and }{\uplambda }_{2}=\frac{\mathrm{M\alpha \zeta T}}{\left(\upzeta +\mathrm{p}\right)\mathrm{d}}.$ Hence ${\mathrm{R}}_{0}$ is the maximum (dominant) of the two eigenvalues of $\mathcal{F}{\mathcal{V}}^{-1}$. Thus we have

$${\mathrm{R}}_{0}=\frac{\mathrm{M\alpha \zeta T}}{\left(\upzeta +\mathrm{p}\right)\mathrm{d}} .$$

Which is the basic reproduction number ${\mathrm{R}}_{0}$ for the System (21). The threshold theorem stated that if the epidemic will not get started unless the initial number of healthy cells exceeds a certain threshold value. i.e.,

If ${R}_{0}<1$ then the disease-free equilibrium is stable.

If ${R}_{0}>1$ the endemic equilibrium exists because the disease spreads throughout the community.

If ${R}_{0}=1$ disease die out.

### Local stability and equilibria

The nonnegative equilibria of Model (21) is ${E}_{0}=({T}_{0},\mathrm{0,0})$,

$${E}^{^{\prime}}=({T}^{^{\prime}},{I}^{^{\prime}},{V}^{^{\prime}}),\mathrm{ where }{T}_{0}=\frac{{T}_{max}}{2\rho }(-(\varpi -\rho )\pm \sqrt{{\left(\varpi -\rho \right)}^{2}+4\frac{\rho }{\gamma } }),$$

$${T}^{^{\prime}}=d\frac{(\zeta +p)}{M\alpha \zeta }, {I}^{^{\prime}}=\frac{1}{\zeta }\left[\gamma -\varpi {T}^{^{\prime}}+\rho {T}^{^{\prime}}(1-\frac{{T}^{^{\prime}}+{I}^{^{\prime}}}{{T}_{max}})\right], {V}^{^{\prime}}=\frac{\alpha \zeta }{d}{I}^{^{\prime}}.$$

The significance of the value ${R}_{0}$ is well-known, which is called as the basic reproduction number. The basic reproduction ${R}_{0}$ is formulated to represent the average number of people who will catch a disease from one contagious host. If we want to understand the nature of transmissible diseases and how disease can spread through a population, we must need to understand the concept of the basic reproduction number. Now we shall look at the geometric features of Model (21) equilibria.

Since ${T}_{0}$ and ${T}^{^{\prime}}$ satisfy

$$\gamma -\varpi {T}_{0}+\rho {T}_{0}\left(1-\frac{{T}_{0}+{I}_{0}}{{T}_{max}}\right)=0,$$

$$\gamma -\varpi {T}^{^{\prime}}+\rho {T}^{^{\prime}}\left(1-\frac{{T}^{^{\prime}}+{I}^{^{\prime}}}{{T}_{max}}\right)=\frac{1}{\alpha \zeta }[\left(\alpha \zeta {T}^{^{\prime}}-d\left(\zeta +p\right)\right].$$

We can get

$$T^{\prime} > d\frac{{\left( {\zeta + p} \right)}}{M\alpha \zeta } \Rightarrow \gamma - \varpi T_{0} + \rho T_{0} \left( {1 - \frac{{T_{0} + I_{0} }}{{T_{max} }}} \right) > 0 \Rightarrow T_{0} > T^{\prime}$$

$$T^{\prime} < d\frac{{\left( {\zeta + p} \right)}}{M\alpha \zeta } \Rightarrow \gamma - \varpi T_{0} + \rho T_{0} \left( {1 - \frac{{T_{0} + I_{0} }}{{T_{max} }}} \right) < 0 \Rightarrow T_{0} < T^{\prime}.$$

Thus if ${R}_{0}>1$, then the positive equilibrium ${E}^{^{\prime}}=({T}^{^{\prime}},{I}^{^{\prime}},{V}^{^{\prime}}$) exists. The Jacobian matrix of Model (21) is follows as:

$$J=\left(\begin{array}{ccc}-\varpi +\rho -\frac{2\rho T+\rho I}{{T}_{max}}-MV& \frac{-\rho T}{{T}_{max}}& -MT\\ MV& -(\zeta +p)& MT\\ 0& \alpha \zeta & -d\end{array}\right).$$

Let ${E}^{*}=({T}^{*},{I}^{*},{V}^{*})$ be any arbitrary state of equilibrium. Then the characteristic equation about ${E}^{*}$ is define as follows:

22 $$\left|\begin{array}{ccc}\lambda +\varpi -\rho +\frac{2\rho {T}^{*}+\rho {I}^{*}}{{T}_{max}}+M{V}^{*}& \frac{\rho {T}^{*}}{{T}_{max}}& M{T}^{*}\\ -MV& \lambda +\zeta +p& -M{T}^{*}\\ 0& -\alpha \zeta & \lambda +d\end{array}\right|=0$$

For equilibrium ${E}_{0}=({T}_{0},\mathrm{0,0})$, (22) reduces to

23 $$\left(\lambda +\varpi -\rho +\frac{2\rho {T}_{0}}{{T}_{max}}\right)[\left( {\lambda }^{2}+\lambda (d+\zeta +p\right)+d\left(\zeta +p\right)- \alpha \zeta M{T}_{0})]=0$$

Hence ${E}_{0}=({T}_{0},\mathrm{0,0})$ is locally asymptotically stable (LAS) for ${R}_{0}<1$.

#### Theorem 5.1

If the basic reproduction number ${R}_{0}<1$, then ${E}_{0}=({T}_{0},\mathrm{0,0})$ is locally asymptotically stable and if ${R}_{0}>1$, then ${E}_{0}=({T}_{0},\mathrm{0,0})$ is unstable.

#### Theorem 5.2.

Let $Q>0$ then for any positive solution $(T\left(t\right), I\left(t\right), V(t)$) of Model (21), $T\left(t\right)\le Q, I\left(t\right)\le Q and V\left(t\right)\le Q$, for all $t$.

#### Proof.

Let ${G}_{1}\left(t\right)=T\left(t\right)+ I\left(t\right)$. Determine the derivative of ${G}_{1}\left(t\right)$ with the solution of the Model (21), we get

$$\begin{aligned} \dot{G}_{1} \left( t \right) & = \dot{T}\left( t \right) + \dot{I}\left( t \right) \\ & = \gamma - \varpi T + \rho T\left( {1 - \frac{T + I}{{Tmax}}} \right) - \zeta I \\ & = - \varpi T - \zeta I + \rho T - \frac{{\rho T^{2} + \rho TI}}{{T_{max} }} + \gamma \\ & \quad \le - hG_{1} \left( t \right) + Q_{0} , \\ \end{aligned}$$

where ${Q}_{0}=\frac{{T}_{max}{\rho }^{2}+4\rho \gamma }{4\rho }$, $h=\mathrm{min}(\varpi ,\zeta )$. Then there exists ${Q}_{1}>1$, depending only the parameters of Model (21), such that ${G}_{1}\left(t\right)<{Q}_{1}$, for all *t.* Then $T(t)$ and $I(t)$ are subsequently bounded above. According to the last equation of Model (21), $V(t)$ has ultimately an upper bound, say, their maximum is $Q$. This completes the proof.

Define $D=\left\{\left(T,I,V\right)\in {R}^{3}:0<T\le Q,<I\le Q, 0<V\le Q\right\}$. Obviously, D is convex.

#### Theorem 5.3.

Suppose that

$${R}_{0}<0;\left(d+ \zeta +p+ \varpi -\rho +\frac{2\rho {T}^{^{\prime}}{I}^{^{\prime}}}{{T}_{max}}+M{V}^{^{\prime}}\right)\left[-\varpi +\rho -\frac{2\rho {T}^{^{\prime}}{I}^{^{\prime}}}{{T}_{max}}\left(d+\zeta +p\right)+ M{V}^{^{\prime}}\left(d+\zeta \right)\right]>0.$$

Then the positive equilibrium ${E}^{^{\prime}}=({T}^{^{\prime}},{I}^{^{\prime}},{V}^{^{\prime}}$), Eq. (23) reduce to

$${\lambda }^{3}+{x}_{1}{\lambda }^{2}+{x}_{2}\lambda +{x}_{3}=0,\mathrm{ where}$$

$${x}_{1}= d+ \zeta +p+ \varpi -\rho +\frac{2\rho {T}^{^{\prime}}{I}^{^{\prime}}}{{T}_{max}}+M{V }^{^{\prime}}>0,$$

$${x}_{2}=(\varpi -\rho +\frac{2\rho {T}^{^{\prime}}{I}^{^{\prime}}}{{T}_{max}})\left(d+\zeta +p\right)- pM{V}^{^{\prime}}>0,$$

$${x}_{3}=d\zeta M{V}^{^{\prime}}>0.$$

We also have

$${x}_{1}{x}_{2}-{x}_{3}=\left(d+ \zeta +p+ \varpi -\rho +\frac{2\rho {T}^{^{\prime}}{I}^{^{\prime}}}{{T}_{max}}+M{V}^{^{\prime}}\right)\left[\left(-\varpi +\rho -\frac{2\rho {T}^{^{\prime}}{I}^{^{\prime}}}{{T}_{max}}\right)\left(d+\zeta +p\right)+ M{V}^{^{\prime}}\left(d+\zeta \right)\right]>0.$$

By Routh-Hurwitz criterion^38^, we have ${E}^{^{\prime}}=({T}^{^{\prime}},{I}^{^{\prime}},{V}^{^{\prime}}$) is LAS.

### Global asymptotic stability (GAS)

This section describes the GAS of the disease steady state. We also established the characteristics for a disease steady state that is GAS.

#### Definition 5.1.

The Model (21) is said to be competitive in D if, for some diagonal matrix $Z=diag({\epsilon }_{1,}{\epsilon }_{2,}\dots {\epsilon }_{n})$ for ${\epsilon }_{i}(i=\mathrm{1,2},3\dots n)$ is either 1 or −1, $Z\frac{\partial f}{\partial x}Z$ has no positive off diagonal elements for all $x\in D.$

#### Theorem 5.4.

The Model (21) is a competitive system.

#### Proof.

By analyzing the Jacobian matrix of Model (21) and selecting the matrix Z as.

$$Z=\left(\begin{array}{ccc}1& 0& 0\\ 0& -1& 0\\ 0& 0& 1\end{array}\right),$$

We can see that, Model (21) is competitive in D in terms of the partial order specified by the orthant $J=\left\{\left(T,I,V\right)\in {R}^{3}:T\le 0, I\le 0, V\le 0\right\}.$ By direct calculation, we get

$$Z\frac{\partial f}{\partial x}Z=\left(\begin{array}{ccc}-\varpi +\rho -\frac{2\rho T+\rho I}{{T}_{max}}-MV& \frac{-\rho T}{{T}_{max}}& -MT\\ MV& -\zeta -p& -MT\\ 0& -\alpha \zeta & -d\end{array}\right)$$

#### Remarks 5.1.

Since D is convex and Model (21) has a competitive in D. The Poincare-Bendixson^39^ property is therefore satisfied by Model (21).

#### Lemma 5.1.

Assume that $n=3$ and D is convex. Suppose Model (21) is competitive in D is convex. And let Model (21) is competitive in D and L is a nonempty compact omega limit set of Model (21). If L contains no equilibria, then L is a closed orbit.

We know that Model (21) has nontrivial periodic orbits from Remarks 5.1 and Lemma 5.1. Let $A$ be a linear operator on ${R}^{n}$ and denote its matrix representation with respect to the standard basis of ${R}^{n}$. Let ${\Lambda }^{2}{R}^{n}$ denote the exterior product of ${R}^{n}$. $A$ include canonically a linear operator ${A}^{[2]}$ on ${\Lambda }^{2}{R}^{n}$ ${u}_{1},{u}_{2} \in$ ${R}^{n}$,define

$$A^{\left[ 2 \right]} \left( {u_{1} \Lambda u_{2} } \right): = A\left( {u_{1} } \right) \Lambda u_{2} + A\left( {u_{2} } \right) \Lambda u_{1}$$

and extend the definition over ${\Lambda }^{2}{R}^{n}$ by linearity. The matrix representation of ${A}^{[2]}$ with respect to the canonically basis in ${\Lambda }^{2}{R}^{n}$ is called the second additive compound matrix of $A$. This is an$$\left(\begin{array}{c}n\\ 2\end{array}\right)$$ $$\left(\begin{array}{c}n\\ 2\end{array}\right)$$ matrix and satisfies the property $(A+{B)}^{\left[2\right]}={A}^{\left[2\right]}+{B}^{[2]}$. In the special case when $n=2$, we have ${{A}^{\left[2\right]}}_{2\times 2}=tr A.$ In general, each entry of ${A}^{\left[2\right]}$ is a linear expression of those of A. For instance, when $n=3$, the second additive compound matrix of $A=({a}_{ij})$ is

$${A}^{\left[2\right]}=\left(\begin{array}{ccc}{a}_{11}+{a}_{22}& {a}_{23}& {-a}_{13}\\ {a}_{32}& {a}_{11}+{a}_{33}& {a}_{12}\\ -{a}_{31}& {a}_{21}& {a}_{22}+{a}_{33}\end{array}\right)$$

Let $\sigma A=\left\{{\uplambda }_{1},{\uplambda }_{2},{\uplambda }_{3},\dots {\uplambda }_{\mathrm{n}}\right\}$ be the spectrum of A. Then $\sigma {A}^{\left[2\right]}=\left\{{\uplambda }_{\mathrm{i}}+{\uplambda }_{\mathrm{j}} 1\le \mathrm{i}\le \mathrm{j}\le \mathrm{n}\right\}$ is spectrum of ${A}^{\left[2\right]}$.

Let $x \mapsto f\left( x \right) \in R^{2}$ be a ${C}^{1}$ function for $x$ in an open set $D\in {R}^{n}$. Consider the differential equation

$$\dot{x}=f\left(x\right).$$

The solution to Model (21) denoted by $x(t,{x}_{0}$) such that by $x\left(t,{x}_{0}\right)={x}_{0}$. A set K is said to absorbing in D for Model (2). if $x\left( {t,K_{1} } \right)[K$ for each compact $K_{1}$ $[D$ and t sufficiently large. We make the following two basic assumptions:

$({H}_{1})$ There exists a compact absorbing set $K[D$.

$({H}_{2})$ Model (21) has a unique equilibrium ${x}^{^{\prime}}$ in D.

If the equilibrium ${\mathrm{x}}^{\mathrm{^{\prime}}}$ is locally stable and all trajectories in D converge to it, it is said to be globally stable in D. If ${\mathrm{x}}^{\mathrm{^{\prime}}}$ is globally stable in D, the assumptions $({H}_{1})$ and $({H}_{2})$ are satisfied. For viral models and many other biological systems with a bounded cone as the feasible region.

$\left({H}_{1}\right)$ is equal to the uniform persistence of Model (21).

#### Lemma 5.2.

A periodic orbit $\Omega =\left\{\Phi \left(t\right);0<t<y\right\}$ of Model (21) is orbitally asymptotically stable with asymptotic phase if the linear system.

24 $${\Upsilon }^{^{\prime}}\left(t\right)=\frac{\partial {f}^{\left[2\right]}}{\partial x}\Phi \left(t\right)\Upsilon \left(\mathrm{t}\right)$$

is asymptotically stable, where $\frac{\partial {f}^{\left[2\right]}}{\partial x}$ is the second additive compound matrix of the Jacobian matrix $\frac{\partial {f}^{\left[2\right]}}{\partial x}$ of $f$.

#### Lemma 5.3.

1. Assume thatassumptions $({H}_{1})$ and $({H}_{2})$ hold
2. Model (21) satisfies the Poincare- Bendix son property.for each periodic solution $x=\Phi \left(t\right)$ to Model (21) with $\Phi \left(0\right)\in D,$ Model (21) is asymptotically stable.
3. $$\left(-1{)}^{n}\mathrm{det}\left({\frac{\partial f}{\partial x}(x}^{^{\prime}}\right)\right)>0.$$
4. Then the unique equilibrium ${x}^{^{\prime}}$ is GAS in D.

## Formulation of the extended HIV model

**Figure 11:**
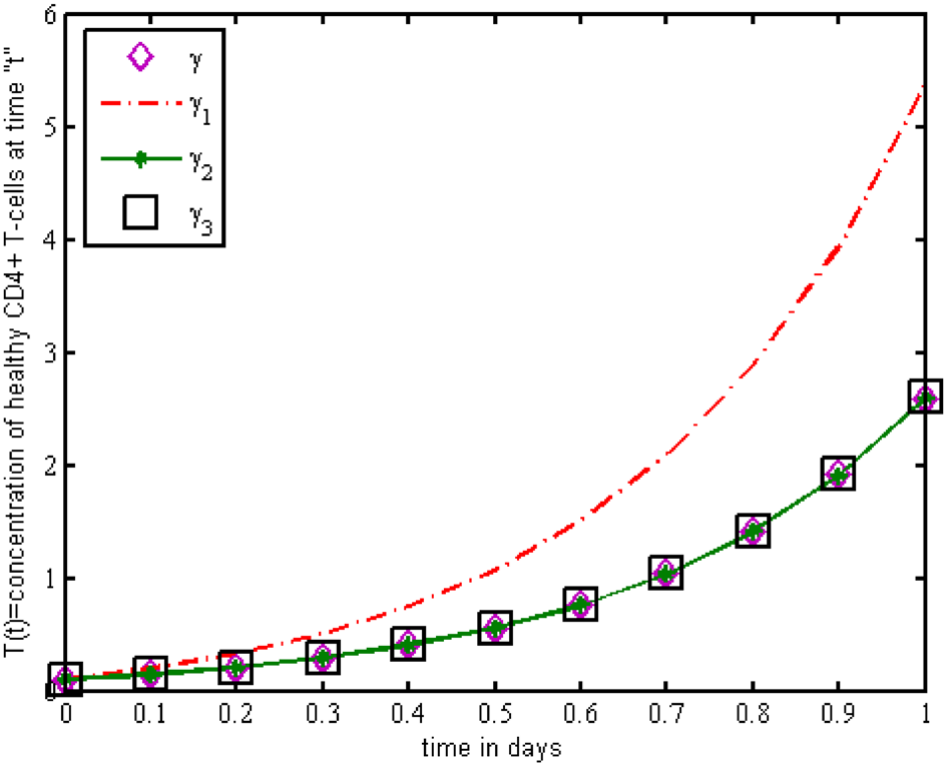
The impact of source terms on $T\left(t\right)$ of HIV infected model.

**Figure 12:**
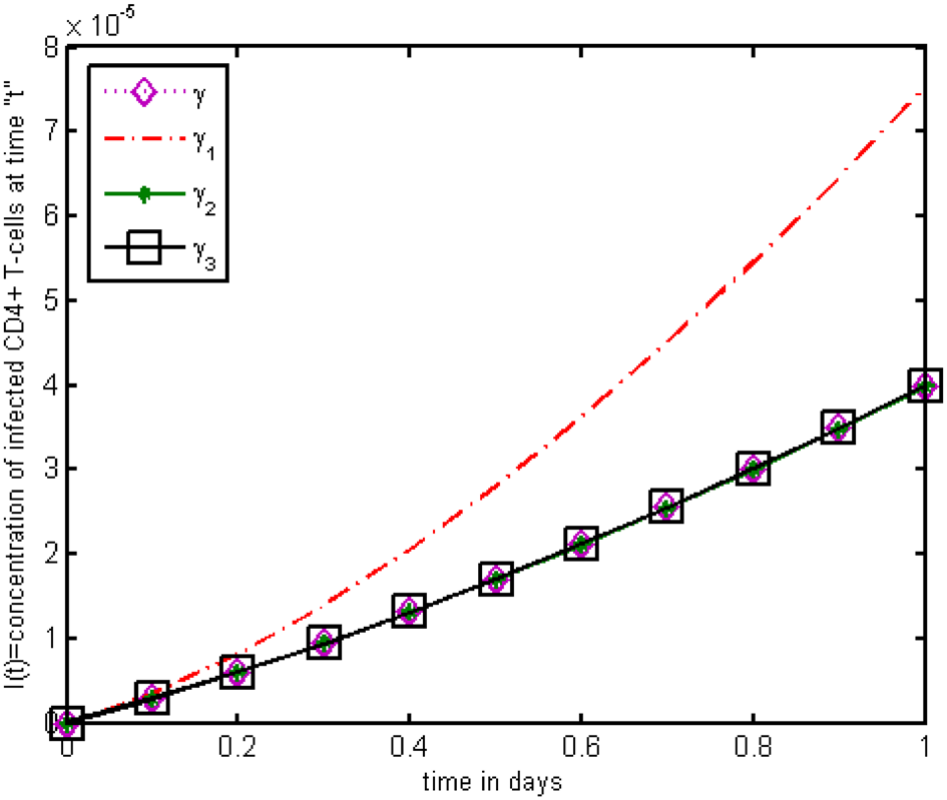
The impact of source terms on $I\left(t\right)$ of HIV infected model.

**Figure 13:**
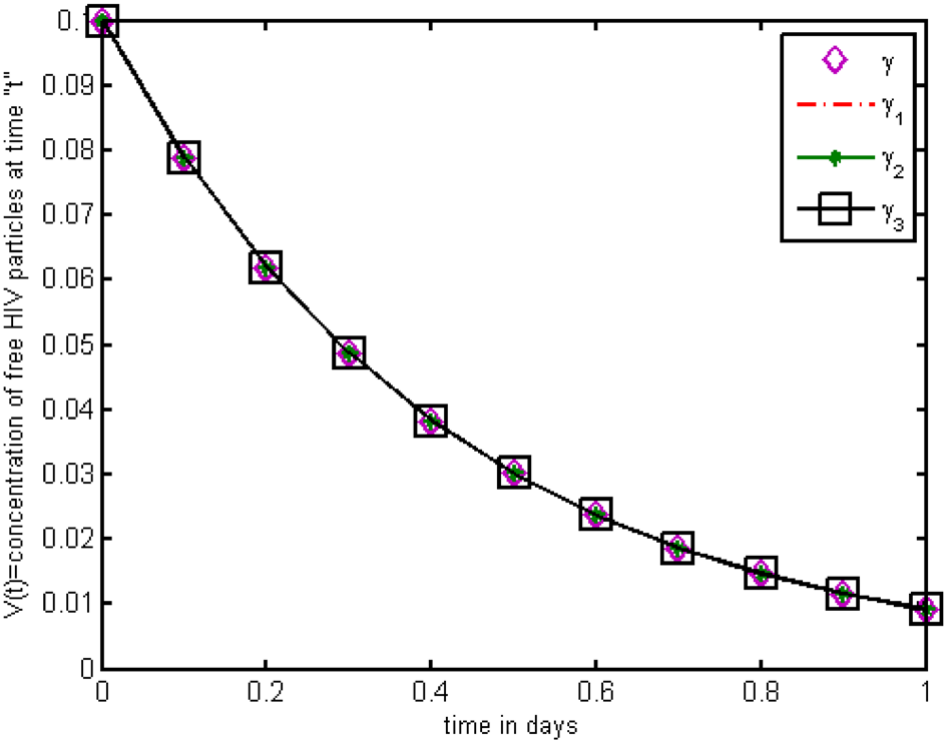
The impact of source terms on $V\left(t\right)$ of HIV infected model.

**Figure 14:**
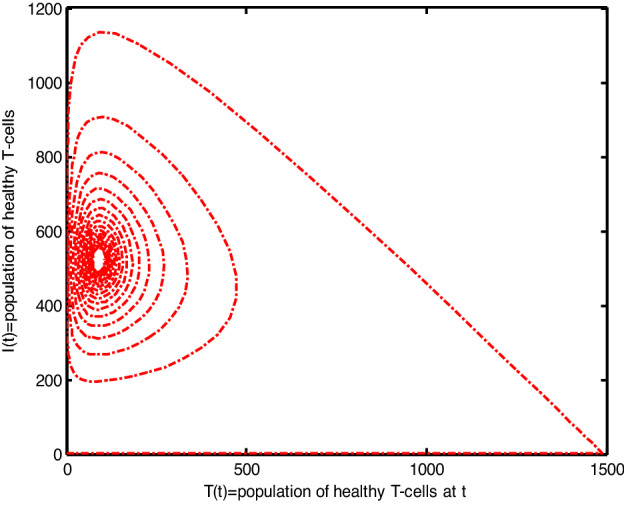
The chaotic behavior of $I(t)$ verses $T(t)$ of the HIV infection model.

**Figure 15:**
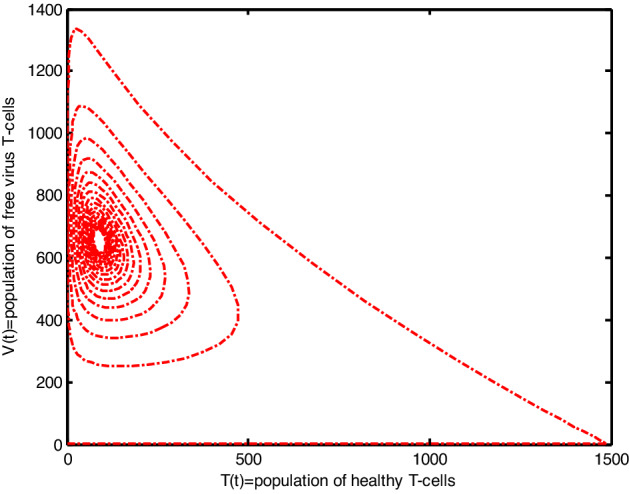
The chaotic behavior of $V(t)$ versus $T(t)$ of the HIV infection model.

**Figure 16:**
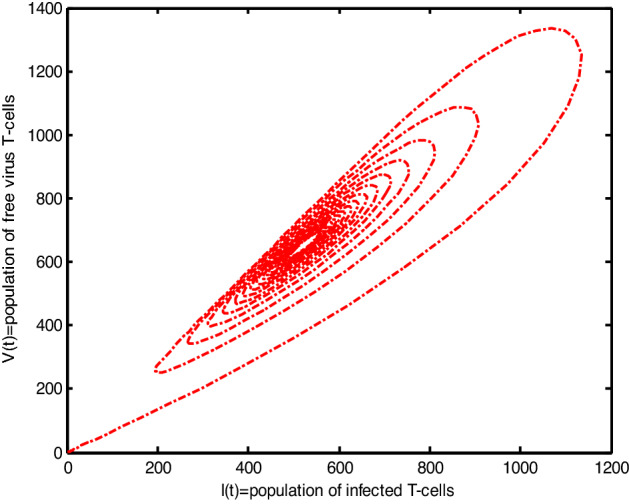
The chaotic behavior of $V(t)$ versus $I(t)$ of HIV infected model.

**Figure 17:**
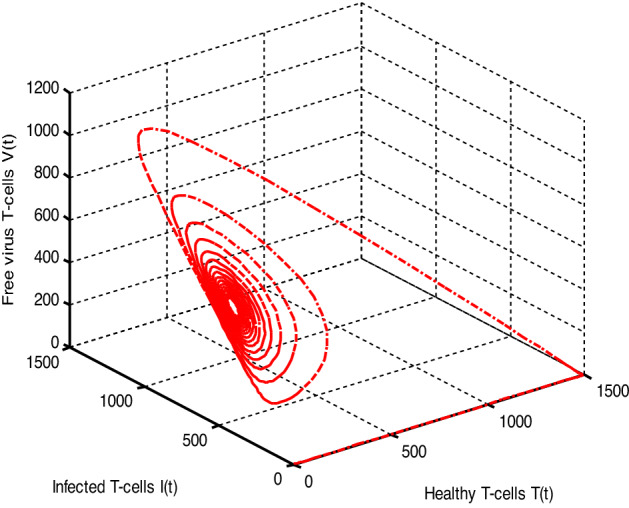
The chaotic behavior of $T(t), I(t) and V(t)$ of HIV infected model.

Throughout the dispersion of HIV infection, several researchers attempted to formulate and solve its epidemic model using a variety of methodologies, analyzing, and comparing their findings to previous findings in order to identify a more effective treatment. In the proposed Model (2), $\gamma$ represents the production of new cells from thymus. The models presented in the literature (see[^28–31^] for details information) based on a stable source term to produce new T-cells. However, these viruses may be capable of infecting T-cells in the thymus and bone marrow after entrance into the human body, leading to a reduced formation of new cells^32^. Therefore, in the current literature, the HIV model (see^32–36^ for details) considered with a nonlinear varying viral load for the formation of new T-cells from the thymus, i.e., Kirschner^33^ and Webb et al.^36^ used the term ${\gamma }_{1}=0.5\gamma +\frac{5\gamma }{1+V(t)}$, Perelson et al.^40^ used ${\gamma }_{2}=\frac{\gamma }{1+V(t)}$, and Perelson^32^ assumed ${\gamma }_{3}=\gamma \mathrm{exp}(-V(t)$. In this paper, we investigated the HIV model outlined above and demonstrated that how varying source depending on viral load affect the dynamical behavior of the improved model. In order to determine solutions of the model, the Galerkin technique is employed. Figures 11, 12, 13 demonstrated that the dynamics of healthy and infected T-cells for ${\gamma }_{1}$ behave differently than $\gamma$, ${\gamma }_{2}$, and ${\gamma }_{3}$ throughout the given period, and that the population dynamics of virus particles significantly exhibit the same dynamics as the stable source term visualized in Fig. 11. Finally, Figs. 14, 15, 16, 17 show the phase diagrams of $I\left(t\right)-T\left(t\right)$, $V\left(t\right)-T\left(t\right), V\left(t\right)-I\left(t\right),$ and $V\left(t\right)-I\left(t\right)-V\left(t\right),$ for HIV-infected model. The graph of each phase has numerically distinct meanings at each stage and does not emphasize on the medical evaluation of solutions.

## Conclusions

In this study, we examined the HIV model, which consists of three nonlinear ordinary differential equations. To solve the model, we used a novel numerical scheme called the continuous Galerkin–Petrov scheme and examined its accuracy and reliability. For comparative analysis, the results of the Galerkin and RK4 schemes are contrasted with those of other conventional techniques, i.e., QL-M, LADM, VLM, MLCM, and MVIM. In addition, we compared the output and absolute errors between the findings of Galerkin and RK4 schemes with the same and different step sizes. After a comparison, it is evident that the suggested scheme produced more accurate and comparable solutions than the solutions of the previously applied schemes for the model. The proposed approach has been proven to be reliable for identifying an approximate solution to real-world situations. After validating the scheme and the MATLAB code, we applied the method to a new model that included the treatment rate. The basic reproduction number is calculated, and the global dynamics of the novel model are determined. It could be observed that the disease-free equilibrium is globally asymptotically stable when it is less than unity and unstable when it becomes greater than unity. On the other hand, we discussed the influence of different non-linear source terms for the production rate of healthy T-cells on the dynamical behaviour of the model. From the observations, we inferred that the patterns of healthy and infected T-cells behave differently throughout the given time and that the population dynamics of virus particles substantially follow the same dynamics as the constant source term. In addition, graphical observations are made to demonstrate the phase diagrams of the mentioned model. The graph of each phase has numerically unique interpretations at each point and is not associated with the medical assessment of solutions. In the future, we plan to apply the suggested Galerkin scheme to other mathematical models in population biology and epidemiology.

## Data Availability

All the data available in the manuscript.
